# Heterologous overexpression of bacterial hemoglobin VHb improves erythritol biosynthesis by yeast *Yarrowia lipolytica*

**DOI:** 10.1186/s12934-019-1231-9

**Published:** 2019-10-15

**Authors:** Aleksandra M. Mirończuk, Katarzyna E. Kosiorowska, Anna Biegalska, Magdalena Rakicka-Pustułka, Mateusz Szczepańczyk, Adam Dobrowolski

**Affiliations:** Department of Biotechnology and Food Microbiology, Wroclaw University of Environmental and Life Sciences, Chełmońskiego 37, 51-630 Wrocław, Poland

**Keywords:** Bacterial hemoglobin, VHb, *Yarrowia lipolytica*, Glycerol, Metabolic engineering

## Abstract

**Background:**

*Yarrowia lipolytica* is an unconventional yeast with a huge industrial potential. Despite many advantages for biotechnological applications, it possesses enormous demand for oxygen, which is a bottleneck in large scale production. In this study a codon optimized bacterial hemoglobin from *Vitreoscilla stercoraria* (VHb) was overexpressed in *Y. lipolytica* for efficient growth and erythritol synthesis from glycerol in low-oxygen conditions. Erythritol is a natural sweetener produced by *Y. lipolytica* under high osmotic pressure and at low pH, and this process requires high oxygen demand.

**Results:**

Under these conditions the VHb overexpressing strain showed mostly yeast-type cells resulting in 83% higher erythritol titer in shake-flask experiments. During a bioreactor study the engineered strain showed higher erythritol productivity (Q_ERY_ = 0.38 g/l h) and yield (Y_ERY_ = 0.37 g/g) in comparison to the control strain (Q_ERY_ = 0.30 g/l h, Y_ERY_ = 0.29 g/g). Moreover, low stirring during the fermentation process resulted in modest foam formation.

**Conclusions:**

This study showed that overexpression of VHb in *Y.* *lipolytica* allows for dynamic growth and efficient production of a value-added product from a low-value substrate.

## Background

Erythritol is a four-carbon polyol produced naturally by some microorganisms as an osmoprotectant. Due to its sweet taste, it is used as a natural sweetener. This polyol occurs naturally in honey, wine and fermented food, is almost non-caloric, and due to its chemical structure it cannot change the level of the insulin in the blood, thus being safe for diabetics. Erythritol has a very low energy level (0–0.2 kcal g^−1^) in comparison to sucrose (4 kcal g^−1^) or to other polyols, yielding approximately 2 kcal g^−1^ [1]. For many years it was believed that erythritol cannot be utilized by the human body, and it is extracted in the urine within 24 h after consumption. However, Hootman et al. observed that about 5% of the consumed erythritol is assimilated by the human cells [2]. On the industrial scale, erythritol is produced from glucose by fermentation of the yeast *Candida magnoliae* or *Moniliella* sp., but it can also be synthesized by the yeast *Yarrowia lipolytica* from glycerol [3, 4].

*Yarrowia lipolytica* is a well-studied unconventional yeast with a huge industrial potential. Due to its high capability for synthesis and storage of fatty acids it is used as a model organism for lipid metabolism in eukaryotic cells [5, 6]. In addition, it can naturally produce organic acids [7, 8] or polyols [9, 10] and it is used as a host for heterologous protein production and non-native chemicals [11]. One of the unique features of *Y. lipolytica* is a capability for utilization of many unconventional carbon sources, such as alkanes, glycerol or fatty acids [12, 13]. Thus the production cost on the industrial scale might be significantly decreased. Moreover, this microorganism possesses GRAS (generally recognized as safe) status; therefore it can readily be used in the food and pharmacy industry [14]. However, despite many advantages, *Y.* *lipolytica* requires a continuous and high oxygen supply, which is a bottleneck in industrial applications.

It has been demonstrated that overexpression of bacterial hemoglobin from *Vitreoscilla stercoraria* (VHb) improves cell growth and production of native and heterologous proteins in many microorganisms [15, 16]. It was suggested that overexpression of VHb improves oxygen diffusion in the host, leading to an improvement in its aerobic metabolism [17]. The influence of the level of the dissolved oxygen (DO) on *Y. lipolytica* metabolism has been widely studied. It was shown, that DO is the most important factor affecting the morphology of this yeast [18]. Moreover, it was shown that next to pH and temperature DO has a significant influence on growth and lipids accumulation [19, 20]. Also high aeration was a crucial factor for effective citric acid synthesis from glycerol-containing waste of biodiesel industry [21, 22]. In the case of ethanol-grown yeasts, the optimum pO2 for citric acid synthesis amounted to 20–60%; a decrease in citric acid production under low aeration (5%) was associated with a drastic decrease in activities of enzymes involved in the TCA and the glyoxylate cycle [23]. Interestingly, the studies performed with sucrose-grown wild-type and the engineered strains of *Y. lipolytica* showed that elimination of oxygen deficiency resulted in increased production of total organic acids up to 145 g/l [24]. Moreover, synthesis of α-ketoglutaric acid from ethanol [25] or rapeseed oil [26] occurs only at a high aeration.

Despite these studies, it has not been tested how VHb overexpression influences *Y. lipolytica* productivity under high osmotic pressure and at low pH, especially when an untypical carbon source such as glycerol is applied. High concentration of glycerol increases osmotic pressure and acts as a stress factor [27].

The aim of this study was to assess the influence of overexpression of the codon-optimized bacterial hemoglobin VHb on growth and production of erythritol by yeast *Yarrowia lipolytica* from glycerol under low-oxygen conditions.

## Methods

### Microorganisms and media

Strains used in this study were *Y. lipolytica* A101 and AJD pAD-VHb. The strains are part of the strain collection of the Department of Biotechnology and Food Microbiology at Wroclaw University of Environmental and Life Sciences, Poland. *Escherichia coli* strains were grown in LB medium (BTL, Poland) according to standard protocols [28]. Yeast Extract Peptone Glucose (YPD) medium was used for the yeast inoculum preparation and contained (g/l): 10 yeast extract (Merck, Germany), 10 peptone (Biocorp, Poland) and 20 glucose (Merck, Germany).

### Bioscreen C

In the Bioscreen C system (Oy Growth Curves Ab Ltd., Finland), the growth of strains was tested. For tests YNB (Yeast Nitrogen Base, Sigma) medium supplemented with glycerol 5% (w/v) was used. The inoculation cultures were grown for 24 h in YPD medium. Consequently, the overnight cultures were centrifuged and the pellets were washed with sterile water. The strains were grown in 100-well plates in 200 μL of YNB medium. The OD_600_ of the cells was standardized to 0.15. Each strain were grown in five repetitions at 28 °C under a constant agitation rate. Growth was monitored by measuring the optical density (OD) at 420–600 nm every 30 min for 48 h.

### Shake-flask experiments and Bioreactor studies

The ability of the engineered strain to produce metabolites was tested in a shake-flask experiment in Erythritol Synthesis Medium (ESM) (g/l): pure glycerol—100; (NH_4_)_2_SO_4_− 2.3 g; MgSO_4_ × 7H_2_O − 1 g; KH_2_PO_4_− 0.22 g; yeast extract—1 g; CaCO_3_– 3 g and distilled water to 1 l. The flask experiment was performed in a 300 ml flask with 30 ml of the ESM medium. The strains were cultivated on a rotary shaker at 28 °C and 180 rpm. The bioreactor study was done in a 5-L stirred-tank reactor (BIOSTAT B-PLUS, Sartorius, Germany) with the working volume of 2.0 L at 28 °C in ESM medium (g/l): pure glycerol—150; (NH_4_)_2_SO_4_− 2.3 g; MgSO_4_ × 7H_2_O—1 g; KH_2_PO_4_– 0.22 g; yeast extract—1 g; and tap water to 1 l. Aeration and stirring rates were set at 0.8 vvm and 500 min^−1^, respectively. pH 3.0 of the medium was maintained automatically by additions of 20% NaOH. The cultures were cultivated until to the depletion of glycerol. The bioreactor with the medium was sterilized in an autoclave at 121 °C for 20 min. All cultures were conducted in three biological replicates and standard deviations were calculated.

### Cloning and transformation protocols

The restriction enzymes used in this study were purchased from FastDigest Thermo Scientific and the digestions were performed according to the producer’s protocols. The PCR reactions done using Phusion high fidelity DNA polymerase (Thermo Fisher Scientific). Ligation reactions were performed using T4 DNA Ligase (Thermo Fisher Scientific) for 10 min at room temperature. The vectors were isolated using the Plasmid Mini Kit (A&A Biotechnology, Poland). Transformation of *E.* *coli* strains was performed using standard chemical protocols. Genomic DNA (gDNA) was isolated from yeast using the Genomic Mini AX Yeast Spin kit (A&A Biotechnology, Poland).

### Construction of overexpression plasmids

A codon-optimized for *Y. lipolytica* gene VHb (461 bp) was cloned into pAD vector [29] using SgsI and NheI sites, resulting in the pAD-VHb plasmid. Next, the plasmid was digested with MssI to form linear expression cassettes lacking of *E. coli* DNA and flanked by *Y. lipolytica* rDNA for targeted integrations. The yeast was transformed according to the lithium acetate method described before [30]. The proper integration of the overexpression cassette was confirmed through gDNA extraction from the obtained colonies and three distinct PCR confirmations.

### RNA isolation and transcript quantification

The strains were grown for 24 h in YNB medium with glycerol (100 g/L). Consequently, cultures were centrifuged for 5 min at 12,000*g*. RNA was isolated using Total RNA Mini Plus kit (A&A Biotechnology, Poland) followed by DNase I (Thermo Scientific, USA) treatment according to the producer’s instructions. RNA quantities were measured using a Biochrom WPA Biowave II spectrophotometer (Biochrom Ltd., UK) equipped with a TrayCell (Hellma Analytics, Germany), next the isolated RNAs were stored in a − 80 °C freezer. cDNA synthesis was proceeded using Maxima First Strand cDNA Synthesis kits for RT-qPCR (Thermo Fisher Scientific). qRT-PCR analyses were performed using the DyNAmo Flash SYBR Green qPCR Kit (Thermo Fisher Scientific) using the Eco Real-Time PCR System (Illumina, USA). The primers qVHb-F (5′-ACCAGCAGACCATCAACATC-3′) and qVHb-R (5′-GCCCATGTCGAATAAAGGTC-3′) bind to the codon-optimized VHb gene, resulting in a 131 bp product. The genes expression level was normalized to the actin gene (ACT-F 5′-GAGTCACCGGTATCGTTC-3, ACT-R 5′-GCGGAGTTGGTGAAAGAG-3′) and analyzed using the ddCT method [31]. Samples were analyzed in three repetitions.

### Analytical methods

The samples (10 ml) taken from the bioreactor cultures were spun down (5 min, 5000 rpm). The pellet was washed with distilled water and filtered on 0.45 μm pore-size membranes. The biomass was determined gravimetrically after drying at 105 °C and it was expressed in grams of cell dry mass per liter (g/l). The concentrations of the metabolites were determined with HPLC using a HyperRez Carbohydrate H^+^ Column (Thermo Scientific, Waltham, MA) coupled to a UV (λ = 210 nm) (Dionex, Sunnyvale, USA) and a refractive index (RI) detector (Shodex, Ogimachi, Japan). The column was eluted with 25 mM of trifluoroacetic acid (TFA) at 65 °C and a flow rate of 0.6 ml min^−1^.

### Calculation of fermentation parameters

The yield of erythritol production from glycerol (Y_ERY_), was calculated using the formula: Y_ERY_ = ERY/GLY and it is expressed in g/g. The productivity of erythritol (Q_ERY_) was calculated by: Q_ERY_ = ERY/t and is expressed in g/l h. “GLY” is the total amount of glycerol consumed (g/l) and “t” is the duration of the fermentation process (h).

## Results and discussion

### Overexpression of codon-optimized VHb in *Yarrowia lipolytica*

For robust growth and efficient production of the metabolites yeast *Y. lipolytica* requires a huge amount of dissolved oxygen in the medium. Previously it was shown that oxygen limitation has a significant impact on fermentation parameters [29] and at oxygen-limiting conditions the fermentation process collapses. This phenomenon is a bottleneck during industrial application, since providing a high amount of oxygen causes foam formation and increases the production costs. Abundant foam formation is undesired in the industry, since it generate a loss of biomass and decreases the productivity, yield and titer of the product.

To address this issue, in this study bacterial hemoglobin VHb was overexpressed in *Y. lipolytica*. In contrast to another study [32] the codon-optimized gene for *Y. lipolytica* was overexpressed in this yeast. It was shown that codon optimization plays a critical role; particularly it can significantly improve heterologous protein expression [33]. *Y. lipolytica* produces erythritol under high osmotic pressure and at low pH. Due to these unfavorable fermentation conditions, this process demands an elevated level of dissolved oxygen. Therefore to increase the intracellular effective dissolved oxygen concentration, the VHb gene was cloned under a hybrid promoter, UAS1B_16_-TEF [34]. Consequently the overexpression cassette excluding the bacterial backbone was transformed into *Y. lipolytica* strain AJD [9], resulting in the strain AJD pAD-VHb. Proper integration into the *Y.* *lipolytica* genome was verified by PCR.

**Fig. 1:**
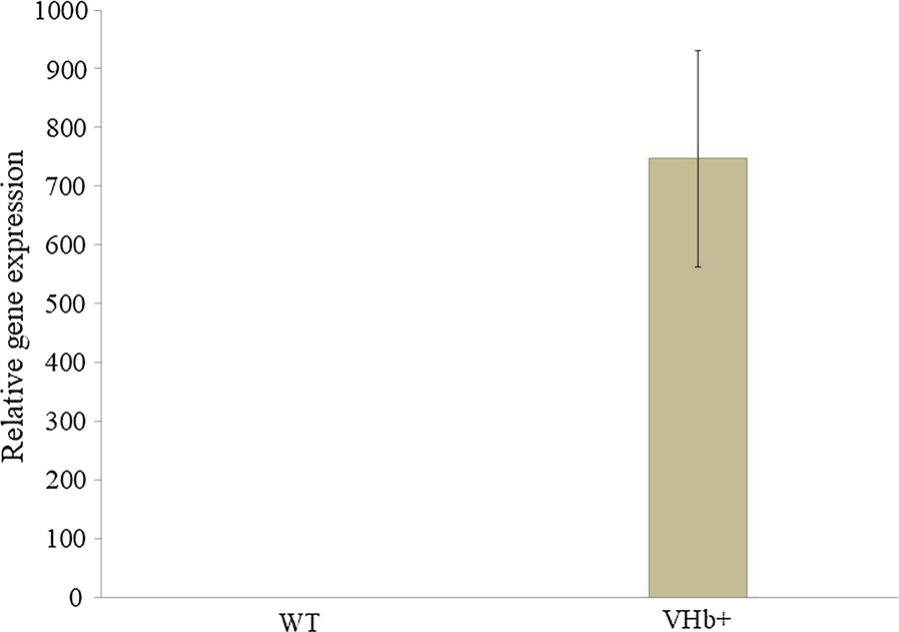
Relative quantification of RNA transcript in the wild strain (A101) and the strain overexpressing VHb (AJD pAD-VHb) using RT-PCR. Actin was used as a reference gene. Strains were grown in YNB/glycerol medium. Samples were analyzed in triplicate and the standard errors were estimated using Illumina Eco software

**Fig. 2:**
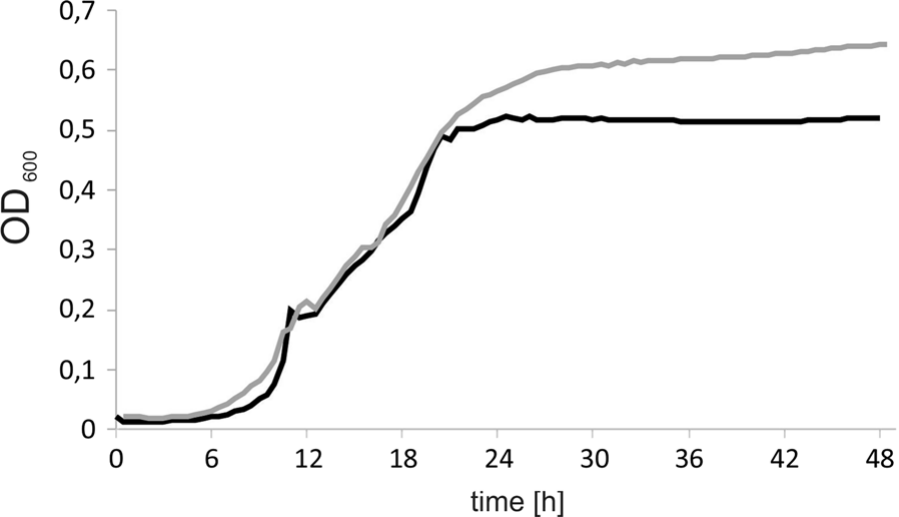
Growth curves of *Y. lipolytica* A101 (black) and AJD pAD-VHb (gray). The strains were grown on a YNB/glycerol medium. Quintuple experiments were performed at 28 °C under constant agitation using Bioscreen C

First, to verify the proper and functional overexpression of the heterologous gene, the level of gene expression was checked via RT-PCR of the total RNA. Therefore, the strains were grown in YNB supplemented with glycerol and the total RNA was isolated after 24 h. In agreement with the assumptions the engineered strain showed high VHb overexpression (Fig. 1), already at the beginning of the process. Thus it was proved that mRNA of VHb was produced by yeast. Such a high gene expression level was previously observed for other heterologous expression under UAS1B_16_-TEF [34]. Next, we sought to test the growth difference between the engineered strain and the wild type under oxygen-limiting conditions in a microplate reader. First, to avoid any negative influence of the low pH and high osmotic pressure, which is required for erythritol synthesis, the strains were grown in YNB medium supplemented with glycerol. As a control the wild-type strain A101 was used. As seen in Fig. 2, the growth rates for the first 18 h were the same for both strains, but after this period, a difference between the strains appeared. The engineered strain showed higher OD than the wild type. It can be explained by the fact that UAS1B_16_-TEF promoter displays the maximum activity after 24 h, which was already observed before [34, 35]. Since the yeast was grown under low-oxygen conditions, the OD for both strains was rather lower than it was observed for the wild type under high-oxygen conditions [36]. Despite this fact, the growth differences between A101 and AJD pAD-VHb strains in this experiment proved that the VHb gene was functionally expressed in the engineered strain, which allowed for good growth of yeast under low-oxygen conditions.

### Erythritol synthesis by the engineered Y. lipolytica strain

**Fig. 3:**
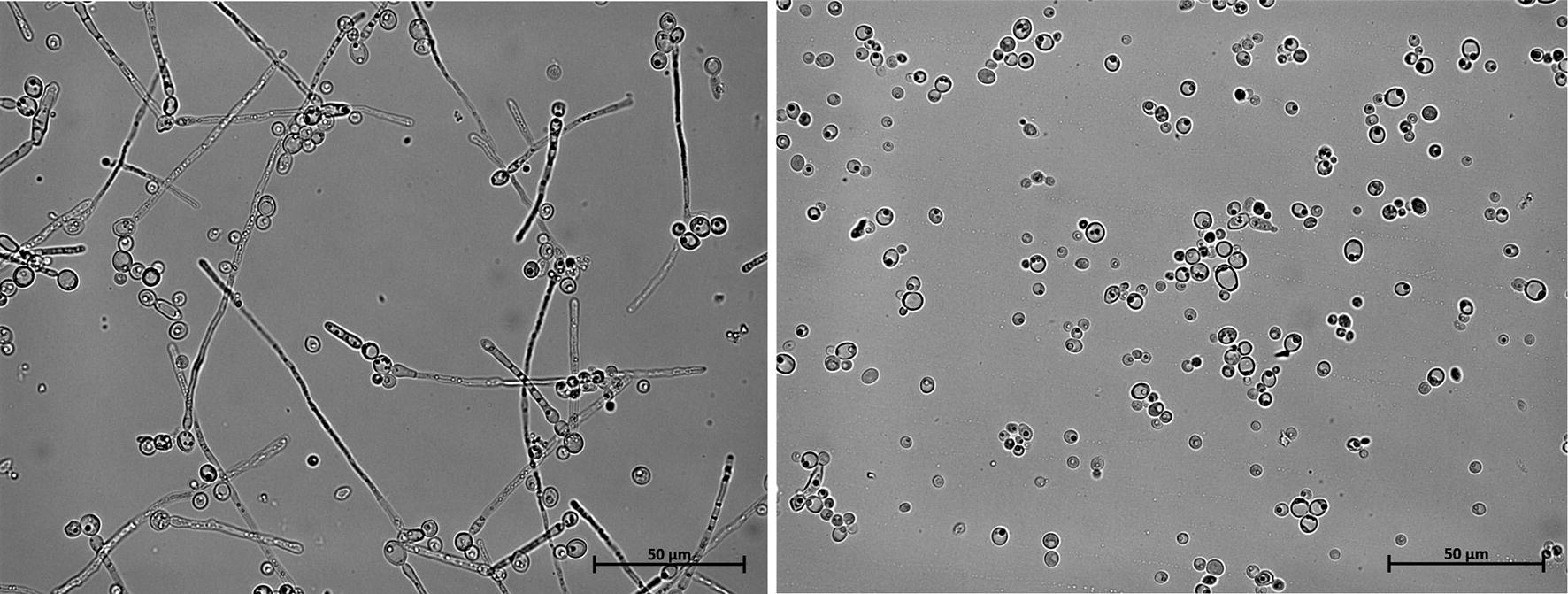
Visualization of *Y. lipolytica* strains A101 (left) and AJD pAD-VHb (right). Strains were grown in the in Erythritol Synthesis Medium. Images were taken at 24 h of cultivation

**Fig. 4:**
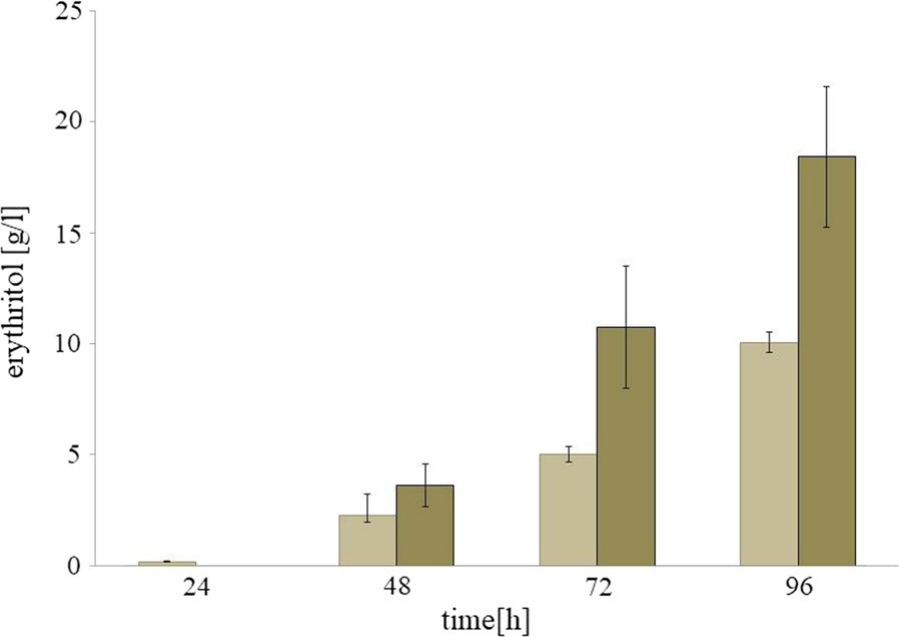
Results of the shake-flask experiment of A101 strain (light brown) and AJD pAD-VHb (dark brown). The cultures were performed in three biological replicates. The error bars represent the standard deviation

Erythritol is produced by the microorganisms in response to high osmotic pressure of the environment. Moreover, in the previous study, it was demonstrated that low pH is favorable for synthesis of this polyol, since production of citric acid is naturally repressed under these conditions [37, 38]. However, these fermentation conditions required high oxygen demand for efficient polyol synthesis [29]; otherwise the process collapses. Thus functional overexpression of the VHb gene in yeast is beneficial for the cell and it supports efficient biosynthesis of the desired metabolites. To test this hypothesis, the shake-flask experiment was performed. The parental strain *Y. lipolytica* A101 was used as a control. The tested strains were grown in medium for erythritol synthesis (for details see Methods) and were incubated at a low speed on the rotary shaker. First, the influence on the cell morphology was tested. In contrast to another study [32], at low oxygen concentrations *Y. lipolytica* strains harboring VHb^+^ showed almost yeast-type cells, whereas the control strain displayed many mycelia-type cells (Fig. 3). It worth noting that yeast-type cells are favorable for the process, since they are more productive than the mycelia-type cells. Interestingly, previously it was shown that at pH 3.0 yeast *Y. lipolytica* forms mostly yeast-type cells, but also the carbon source has an influence on the cell type [29, 39]. However, when an additional stress factor was added (low level of dissolved oxygen), at high osmotic pressure and low pH, the wild-type *Y. lipolytica* strain forms ca. 50% of the cell as mycelium (Fig. 3). Similar results were obtained before, during production of threo-Ds-isocitric acid at low aeration (pO2 5–10%) formation of pseudomycelial forms was induced [26]. The advantage of the yeast-type cell for production of erythritol is confirmed by the results of the shake-flask experiment. For the first 24 h of the cultivation, the wild-type produced more erythritol than the VHb^+^ strain. However, when the UAS1B_16_-TEF promoter achieved the highest activity, the difference between the strains was reversed. The engineered strain started to produce more erythritol and consequently after 96 h of cultivation produced 18.42 ± 3.2 g/l, whereas the control strain produced only 10 ± 0.5 g/l (Fig. 4). The engineered strain showed almost twice as high productivity (Q_ERY_ = 0.19 g/l h) than the wild-type Q_ERY_ = 0.1 g/l h. The engineered strain obtained similar erythritol titer as the wild-type A101 under high-oxygen conditions [40]. Biomass produced by the parental strain A101 was slightly higher (8.8 g/l) than the engineered strain (7.2 g/l). Interestingly, in contrast to a previous study [29], the modified strain produced a small amount of citric acid (3.43 ± 1.1 g/l), slightly more than the control (1.33 ± 0.36 g/l). Also synthesis of arabitol and mannitol varied at a level below 2 g/l (Table 1). The high selectivity during production of the desired metabolite is a benefit during industrial application, since it reduces purification costs.

**Table 1:** Metabolites and biomass production by *Y. lipolytica* strains at the end of the shake-flask experiment (96 h) and bioreactor experiment (144 h) under oxygen limitation conditions

Strain	Erythritol [g/l]	Arabitol [g/l]	Mannitol [g/l]	Citric acid [g/l]	Biomass [g/l]
Shake-flask experiment					
A101	10.06 ± 0.5^a^	1.26 ± 0.1	0.53 ± 0.05	1.33 ± 0.36	8.80 ± 1.2
AJD pAD-VHb	18.42 ± 3.2^a^	1.32 ± 0.6	1.55 ± 1.6	3.43 ± 1.1	7.20 ± 0.3
Bioreactors experiment					
A101	44.20 ± 3.5^b^	1.60 ± 0.3	3.95 ± 0.2	17.85 ± 7.4	32.20 ± 2.5
AJD pAD-VHb	55.75 ± 4.2^b^	1.75 ± 1.1	3.70 ± 2.4	23.75 ± 11.2	28.80 ± 3.5

^a^Statistically significant at p ≤ 0.05

^b^Statistically significant at p ≤ 0.1

Due to promising results, a large-scale experiment was performed using a 5-L bioreactor to test the conditions for robust erythritol production under oxygen-limiting conditions.

### Scale-up process of erythritol synthesis at low oxygen concentrations

As mentioned before, during cultivation of yeast on an industrial scale, a crucial issue is providing a sufficient amount of the oxygen to the medium. This process requires intense agitation, which is undesirable because it causes foam formation and increases costs of energy consumption. The previous experiment proved that overexpression of bacterial hemoglobin VHb in yeast *Y.* *lipolytica* allows for good cell growth and efficient erythritol synthesis under low-oxygen conditions. Given these results, we sought to cultivate the AJD pAD-VHb strain in a bioreactor, to test production of erythritol by the engineered strain on a larger scale. To decrease the oxygen level, the fermentations were conducted at lower agitation (500 rpm) than that applied in the standard procedures (800 rpm) as described previously [29]. The aeration was fixed at 0.8 L/min, and again as a control strain A101 was used.

**Fig. 5:**
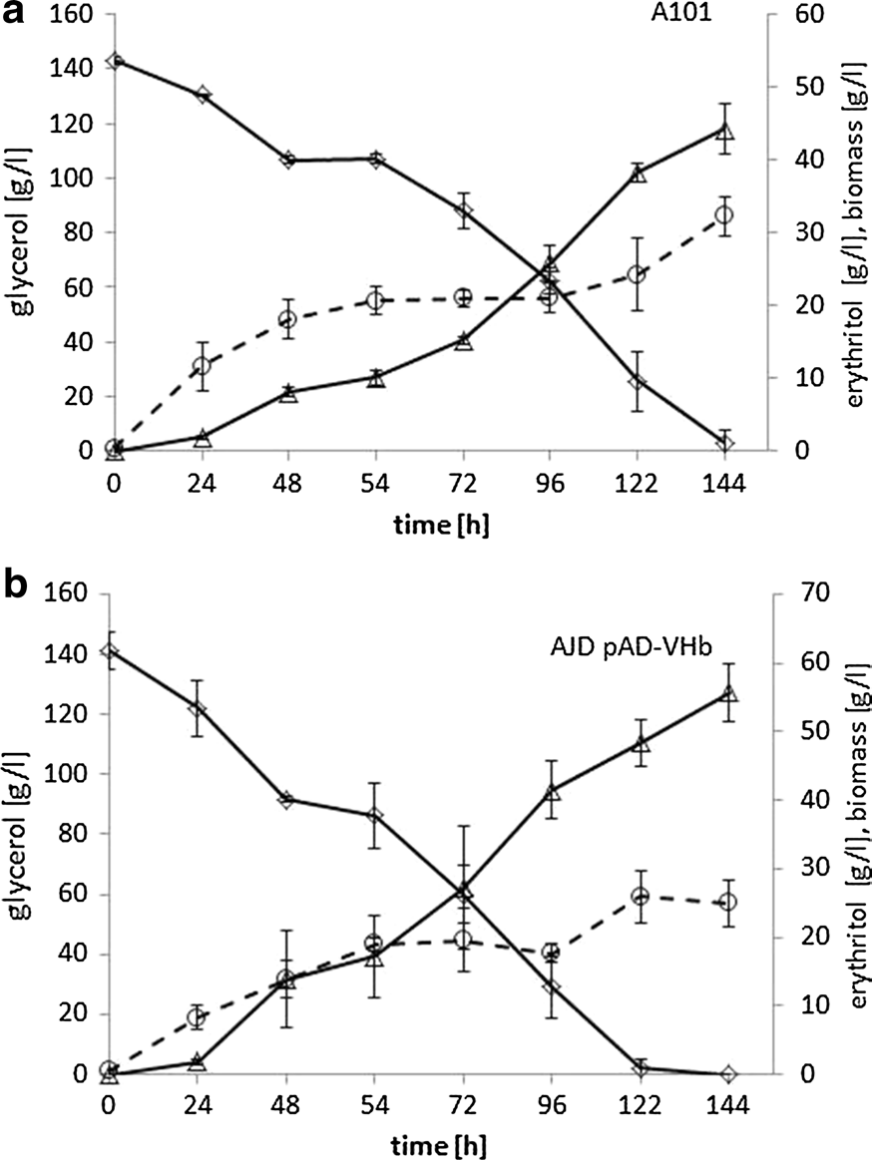
Batch bioreactor fermentations with the control strain A101 (**a**) and strain overexpressing VHb (**b**). The cultures were performed in three biological replicates. The error bars represent the standard deviation

Dynamic growth of *Y. lipolytica* caused that during the first 8 h of growth, the dissolved oxygen fell to below 1% during the bioreactor study. This phenomenon was observed before during another study [41]. However, the strain harboring VHb^+^ could easily grow and efficiently produced erythritol under these conditions (Fig. 5). Biomass of the AJD pAD-VHb strain reached 28.8 g/l. This is a high titer of biomass in erythritol synthesis medium, for the reason that its paternal strain A101 is known for robust biomass production [42]. As seen in Fig. 5, the biomass of strain A101 reached 32 g/l at oxygen-limiting conditions, but the final erythritol titer was significantly lower. Again, for first 24 h production of erythritol for both strains was the same, but the engineered strain utilized glycerol more rapidly and this trend was maintained to the end of the fermentation. After 48 h of the process, the strain AJD pAD-VHb started rapid synthesis of erythritol. It produced 55.75 g/L of erythritol within 144 h, resulting in Q_ERY_ = 0.39 g/L h and Y_ERY=_0.37 g/g. The control strain A101 produced 44.2 g/l of erythritol and within 144 h it did not utilize the total amount of glycerol, with the values Q_ERY_ = 0.31 g/L h and Y_ERY_ = 0.29 g/g. Interestingly, production of arabitol and mannitol was significantly inhibited in comparison with the shake-flask experiment, and the maximum content of each side-metabolite did not exceed 4 g/L. Unexpectedly, citric acid production was significantly increased for both strains (Table 1). This was an astonishing result, since *Y. lipolytica* was known to be unable to produce high quantities of citric acid at low pH. This might be explained by the oxygen-stress condition, under which the carbon flux was redirected into the TCA cycle, resulting in increased citric acid synthesis. A similar effect of the enhanced citric acid synthesis for the wild-type strain was observed previously [8]. During that study the A101 strain was cultivated on crude glycerol, and consequently a high osmotic pressure was a stress factor.

For further optimization of erythritol synthesis at oxygen-limiting conditions also other strategies might be employed. Metabolic engineering combined with adaptive laboratory evolution (ALE) might significantly improve the capabilities of the host strain for synthesis of this polyol. It was shown that various strategies of ALE improve lipid storage by yeast *Y.* *lipolytica* [43]. For erythritol production cell morphology might be use as a selective factor for artificial selection. However, this interesting topic requires more detailed research.

In this study the results showed that overexpression of VHb in yeast *Y.* *lipolytica* allows for efficient growth under low-oxygen conditions and it is a good starting point for further studies for production of the desired metabolites from various carbon sources.

## Conclusions

The study showed that functional overexpression of the codon-optimized bacterial hemoglobin VHb in yeast *Y. lipolytica* allows for robust growth of yeast on glycerol and efficient synthesis of erythritol. The engineered strain produced 55.75 g/L of erythritol during 144 h of fermentation at low oxygen concentrations. Moreover, the engineered strain showed almost only yeast-type cells at low pH, which is beneficial for production of the desired metabolites on the industrial scale, where the oxygen supply is hindered. Low agitation during the fermentation process avoids foam formation, which is a significant issue of the biotechnology industry. In summary, overexpression of VHb combined with modification of growth conditions allows for synthesis of a value-added product from low-cost feedstock.

## Data Availability

The authors promise the availability of supporting data
